# Persistence of commuting habits: context effects in Germany

**DOI:** 10.1007/s00168-023-01223-4

**Published:** 2023-05-26

**Authors:** Ramona Jost

**Affiliations:** Institute for Employment Research (IAB), Regensburger Str. 104, 90478 Nuremberg, Germany

**Keywords:** J60, R10, R19, R23

## Abstract

In this study, I investigate the commuting behavior of workers in Germany. Using comprehensive geo-referenced administrative employee and firm data, I can calculate the exact commuting time and the distance between workers’ residence and workplace locations. Based on a behavioral economic approach (Simonson and Tveresky in J Mark Res 29:281–295, 1992), I show that individual commuting decisions are influenced by wages and individual heterogeneity as well as depending on the context individuals observed in the past. In particular, my results show that previously observed commutes have an impact on subsequent commuting behavior: workers choose longer commuting times in the region they recently moved to when the average commute in the region they left was longer. The results indicate that while selectivity and sorting do not influence the effect of the context, the inclusion of individual fixed effects is crucial.

**Supplementary Information:**

The online version contains supplementary material available at 10.1007/s00168-023-01223-4.

## Introduction

The importance of commuting is growing rapidly—both the number of commuters and the distance they commute are growing steadily (Gimenez-Nadal et al. 2020). From an economic perspective, commuting is essential for a well-functioning labor market as it is an important measure to overcome spatial separations (Lux and Sunega 2012; Zabel 2012). At the individual level, commuting implies better labor accessibility and subsequently improves job and career opportunities, leading to better outcomes and improved individual utility. However, commuting also has negative impacts on both the environment and the infrastructure (Brueckner 2000; Rouwendal and Rietveld 1994), as well as on individuals’ well-being as it is associated with congestion and high costs (Frey and Stutzer 2007). Understanding the determinants of and the reasons for commuting is thus an important topic for policymakers dealing with economic and labor market issues.

Studies on commuting find different factors and effects that influence individuals’ commuting behavior, for example commuting is more common among males and among workers with higher incomes as well as among homeowners. The same applies to workers who are older and work in specific occupations and have specific skill levels (Gimenez-Nadal et al. 2018, 2020; Ross and Zenou 2008; Hanson and Johnston 1985; Dargay and Clark 2012; McQuaid and Chen 2012).

However, individuals’ commuting behavior might also be explained from a behavioral economic perspective. In particular, previous research shows that previously observed options can influence individuals’ perceptions and therefore their subsequent decision-making behavior (Simonson and Tveresky 1992). Applied to individuals’ commuting behavior this means that previously observed commuting options influence their preferences for commuting and consequently their own commuting decisions. This approach can explain, for example, why individuals who move to Munich commute 30 percent less than the average in Munich if they come from regions with shorter average commuting times, while individuals commute 35 percent more than the average in Munich if they previously lived in regions with longer commuting times than those typical in Munich. This might indicate that commuting decisions are influenced by the context of commuting options observed in the past, such as other individuals’ commutes.

This study analyzes such commuting behavior, based on the study conducted by Simonsohn (2006) for the US, and contributes to the literature in at least four ways: first, it contributes to the literature on commuting behavior and the factors that are important for explaining commuting (Gimenez-Nadal et al. 2020; Dargay and Clark 2012; McQuaid and Chen 2012). In particular, I show that the context of commuting options observed in the past is crucial for analyzing individuals’ commuting behavior. In this context I show that the results obtained by Simonsohn (2006) are biased due to the omission of individual fixed effects and the consideration only of migrants between two metropolitan areas. Second, I reveal effects for different groups, discussing effect heterogeneity for age, gender, skill level, as well as rural and urban areas, for an entire country. Third, I use geo-referenced employer-employee data. These administrative registry data possesses higher validity than survey data and provides precise information about individuals’ residence and workplace locations with a high number of observations. This makes it possible to calculate the exact commuting distance and time for German workers. Fourth, the study contributes to the migration literature (van Ham and Hooimeijer 2009; Brueckner and Stastna 2020; Shuai 2012). In particular, I show that the greater the difference between a worker’s individual commuting time and the average commuting time at their place of residence, the more likely they are to move again.

When individuals choose where to live, they face the difficult decision of how far they are willing to commute, weighing up the benefits and costs of commuting. Advantages of commuting may include cheaper rents and housing prices outside the city center, resulting in a higher disposable income. Furthermore, commuting can provide more job opportunities for individuals who live in rural areas where there may be no or no adequate employment offers. However, commuting also has disadvantages; it takes up time, causes stress, and impacts the reconciliation of work and family. It can therefore have a negative effect on individuals’ well-being (Frey and Stutzer 2007). When deciding how far they wish to commute, individuals have to trade off the benefits with the disutility of commuting. Indeed, costs and benefits do not have the same effect on utility: the response to losses is stronger than the response to the corresponding benefits (loss aversion, Kahneman and Tveresky 1979). In the context of commuting decisions, however, Dauth and Haller (2020) find no sign of loss aversion, which contradicts previous experimental evidence (Tveresky and Kahneman 1991).

Empirical evidence from urban economics reveals the disutility of commuting for which individuals wish to be compensated. For the Netherlands, van Ommeren et al. (2000) and van Ommeren (2005) find a marginal willingness to pay for an additional kilometer of commuting of 0.15 euros per day or 17 euros for one additional hour of commuting (van Ommeren and Fosgerau 2009). With regard to compensation by the employer, Heuermann et al. (2016) find that employers compensate only few employees directly for additional commuting costs. Hence, the decision to commute is mainly an individual one, which can be strongly influenced by prior experiences.

However, individuals are often unable to assess correctly the disutility of commuting and are frequently uncertain about their preferences, which contradicts the standard economic theory (Kahneman and Tveresky 1979). Instead, they form their preferences as and when they are needed, for instance when making choices (Bettman et al. 1998). For example, in the context of commuting decisions, individuals rely on a wide range of possible cues, such as other individuals’ commutes. Moreover, in the literature on decision-making (Bettman et al. 1998; Huber et al. 1982) it becomes fundamental that an individual’s decision can be influenced by the context: individuals interpret information by comparing it not only to other available options, but also to what was recently observed. According to Hartzmark and Shue (2017), these context effects have the potential to affect a variety of important real-world decisions. They not only distort judicial perceptions of the severity of crimes, leading to unfair sentencing, but also affect employee hiring, medical diagnoses as well as housing and commuting decisions.

The context effect that is relevant for this study is the background context effect, according to which choices depend on options encountered in the past—preferences can change with the history of choices. The intuition behind this is that the same product may seem more attractive against the background of less attractive alternatives and unattractive compared to more attractive alternatives (Simonson and Tveresky 1992). Simonson and Tveresky (1992) document this effect in an experiment comprising two stages in which subjects have to make choices in sequence. In the first stage, half of the subjects are confronted with two options that have a relative high cost for one attribute, and the other half should make a choice with a relatively low cost for the same attribute. In the second stage, all subjects are confronted with the same choice. In line with the background context effect, subjects who are confronted with a relatively high cost for an attribute in the first stage are more likely to choose the more expensive option in the second stage because it appears cheaper to them.

There is ample evidence of the background context effect. Bhargava and Fisman (2014) demonstrate this effect in the context of speed dating. They show that the attractiveness of previous partners reduces the probability of finding a date. Moreover, Hartzmark and Shue (2017) demonstrate that today’s earnings impress investors more when previous earnings were poor. Furthermore, Simonsohn and Loewenstein (2006) present the effect with regard to housing choices: individuals who move from cities with relatively high housing costs are more likely to pay higher prices in the new city compared to individuals coming from cities with cheaper markets. Applied to commuting behavior, this means that commuting options encountered by individuals in the past affect their current commuting decisions. However, relatively little research has been conducted into when and why the background context effect influences commuting decisions. The only such study was conducted by Simonsohn (2006). He considers individuals relocating between two metropolitan areas in the US and takes the average commuting time in the previous city as a proxy for commuting options encountered in the past to examine how previously observed commutes influence commuting decisions when moving to a new city. He finds that individuals choose longer commutes in the new city, the longer the average commute was in the city they came from. Commuting decisions are thus influenced by commuting options encountered by individuals in the past, which is in line with the background context effect.

In this study I consider workers who relocate between NUTS-3 regions in Germany and examine the context effect for workers of an entire country, which is why I deviate from the approach of Simonsohn (2006) and use the average commuting time at the NUTS-3 level for the proxy of commuting options encountered in the past. The results show that individuals coming from backgrounds with longer average commuting times initially choose longer individual commutes in the destination region compared to individuals from regions with shorter average commutes.

In contrast to Simonsohn (2006), I additionally differentiate between individuals moving between different region types of rural and urban regions and thus I show that the context effect is strongest for workers who move from rural to urban areas.

Further, the robustness checks show that selectivity of a relocation does not influence the effect of the context and I find no evidence of workers selecting themselves into regions because of their taste for commuting. However, my results do indicate that it is very important to control for individual fixed effects. Moreover, I find no sign of stable taste difference as traditional economic theory would suggest.

The remaining paper is structured as follows. Section 2 provides the theoretical motivation for the background context effects. Section 3 discusses the data and the sample. The identification strategy used is shown in Sect. 4. The empirical results are presented in Sect. 5, and Sect. 6 concludes.

## Theoretical motivation for the background context effects

As empirical evidence shows, decisions are preference-dependent (Bettman et al. 1998; Huber et al. 1982; Hartzmark and Shue 2017; Bhargava and Fisman 2014; Simonsohn and Loewenstein 2006). However, these preferences change with previously observed options. As Tveresky and Simonson (1992) demonstrate in their background contrast experiment, individuals’ previous experiences influence their perceptions and therefore their subsequent decision-making behavior. For commuting decisions, this implies that commuting options encountered previously affect current commuting preferences and thus individuals’ commuting behavior. The following approach is based on this concept, which is also used by Simonsohn (2006). The idea is that the disutility of commuting decreases when a person was only confronted with longer commuting options in the past, whereas, the disutility increases when individuals were only exposed to short commutes.

To investigate this approach and to measure the effect of the context, I use relocations involving individuals moving between two NUTS-3 regions in Germany. According to the background contrast experiment conducted by Tveresky and Simonson (1992), the commuting behavior after the move should be affected by previously observed commuting options. This concept is formally represented as:

1 $$\alpha_{t}^{*} = \left( {1 - \beta } \right)\alpha_{t - 1} + \beta \left( {\alpha_{t} } \right)$$

with ${\upbeta } \in \left[ {0,1} \right].{ }$ Abstracting all other influences, such as sociodemographic factors, $\alpha_{t}^{*}$ represents a person’s individually chosen commuting time as a weighted sum of the observed commuting options in the present $\alpha_{t}$ and the past $\alpha_{t - 1}$, with the weights decreasing exponentially into the past (Ryder and Heal 1973). More precisely, under the assumption of *β* = 1 there is no impact of commutes observed in the past on the current commuting time, since $\alpha_{t}^{*}$ = $\alpha_{t}$ and thus no impact of the context. In contrast, if *β* = 0 the current commuting preferences are determined only by the previously observed commuting times, corresponding to $\alpha_{t}^{*}$ = $\alpha_{t - 1}$. In the following, I expect *β* to take values between 0 and 1 (0 < *β* < 1), such that two otherwise identical individuals with different numbers of previously observed commuting options will have different levels of $\alpha_{t}^{*}$ when moving to the same region. Moreover, I use the average commuting time in the region of residence before the move as a proxy for previously observed commuting options (Simonsohn 2006)^1^. According to Eq. (1), individuals moving from regions with longer average commutes accept a longer commuting time $\alpha_{t}^{*}$ when choosing places of work and residence in the destination region compared to individuals coming from regions with shorter average commuting times. This is the first prediction I investigate in this study.

**The average commuting time in the region a person leaves has a positive influence on the individually selected commuting time in the destination region**

However, if individuals stay in the new region and observe the commuting options in the new region, their preferences for commuting change due to the new observed commutes in the new region. This leads to a change in the desired commuting duration. For example, movers who relocate from regions with longer commutes to regions with shorter ones initially have a greater tolerance for long commutes and prefer cheaper and larger living space outside the city center. Therefore, they initially commute longer than the average commute in the new region. If they remain in this region and observe shorter commutes, however, their preferences for shorter commutes grow and the disutility for commuting increases. They thus become dissatisfied with the commutes they chose initially and might move again within the new region to reduce their commuting time, thereby correcting an originally excessive amount of commuting. This relationship is illustrated by the second prediction.

**Individuals readjust their commuting times and move again when remaining in the new region**

The second prediction is therefore useful for ruling out explanations based on stable unobserved differences across individuals who move from different regions. Because if individuals who come from regions with longer average commutes travel more after relocating because they are different from those coming from regions with shorter average commutes, I would not expect them to revise their commutes by moving again.

## Data and sample selection

### Data

For the analysis, I use the employment biographies of a 6-percent random sample of all German workers subject to social security contributions. The administrative registry data does not include self-employed persons or civil servants; however, it covers more than 80 percent of the German labor force. The Employment History (BeH – Beschäftigenhistorik V10.01.00, 2016) collated by the Institute for Employment Research (IAB) provides exact information about periods of employment based on the status reports submitted to the pension insurance. Besides the sociodemographic characteristics, information at the firm level are included, which comes from the Establishment History Panel (BHP). This dataset contains information about the branch of industry, the establishment location, number of employees and marginal part-time employees. As daily wages are top-coded at the social security contribution ceiling, I use the imputation procedure developed by Card et al. (2013) to recover wages above this threshold.

A unique feature of this dataset is the supplement IEB GEO, which provides anonymized address information in the form of geocodes for the locations of an individual’s residence and place of work for the years 2000–2014 (Ostermann et al. 2022). Combining this address information with road network data from OpenStreetMap, I calculate door-to-door commuting distances (Huber and Rust 2016; Dauth and Haller 2020; Duan et al. 2022). It is only possible to determine distances for individuals traveling by car in this way; those for users of public transport may differ. However, the car is the most important mode of transport. Almost 70 percent of workers commute to work by car (Destatis 2017), whereas only 14 percent of commuters use the public transport system.^2^ In addition, to calculate the commuting time I take average values for highways, primary, and residential roads. By using geocodes, the commuting time is not limited by administrative units, which reduces measurement error for individuals close to administrative borders and mitigates the problem of spatial sorting within areas. Yet, using driving time can cause issues regarding the experienced commuting time: for example, the algorithm cannot recognize dense traffic in the daily rush hours. Nevertheless, as the time is measured before and after the regional move, the change in the duration might be affected less by this measurement problem.^3^

### Sample

In this study, I investigate the commuting behavior of German workers, excluding persons in marginal and part-time employment as well as workers older than 57 and younger than 18 years of age. Regarding the commuting time, I restrict the sample to workers with a commuting time between 1 and 90 min. I choose 1 min as the minimum because this represents the first percentile of the data and hence ensures that outliers who do not commute are not considered. The restriction to 90 min is because the data does not provide any information about the number of commuting trips. Thus, the data could also include workers who commute weekly and have a second place of residence. To exclude those workers, I restrict the data to workers with commuting times of up to 90 min. This is comparable to other German studies that restrict the commuting distance to 100 km (Dauth and Haller 2020; Duan et al. 2022) and ensures that commuting is conducted on a daily basis.

To test prediction 1, whether the average commuting time in the region a person leaves has a positive influence on the individually selected commuting time in the destination region, several restrictions have to be considered. First, to be able to analyze commuting decisions, I have to consider only those individuals who face such a decision. This group comprises individuals who are required to make a new commuting decision due to moving home or changing their job. For my study, however, I consider individuals who simultaneously change both their place of residence and their place of work. The reason for this is, first, that for individuals who only change their place of work it is not possible to examine the influence of the context of commutes observed in the past, because for job changers the region of the place of residence does not change.^4^ Second, if individuals only change their place of residence they might, for example, be relocating due to dissatisfaction with commuting and I would therefore not be able to identify the influence of the context correctly.^5^ To avoid this, I restrict the sample to workers who change both residence and workplace locations, which further guarantees a relocation of the entire center of their lives. In addition, I restrict the sample to those movers who relocate between two of the 402 German NUTS-3 regions.^6^ I also keep the NUTS-3 region of the place of work and the place of residence constant for two years before and after the move. This guarantees that movers are able to adopt the commuting options as well as the commuting behavior of the region they lived in. In addition, this assumption means that it is possible for movers to relocate again within the target region to readjust their initially chosen commuting time. After these restrictions I identify 15,671 workers who move between two NUTS-3 regions. Furthermore, the time periods are categorized to *t* − 1 for the year before the move, *t* = 0 for the year of the relocation and *t* + 1 for the year after the move.

To test prediction 2, I look at workers who relocate again within the new region in period *t* + 1 (one year after the move), keeping the place of work constant. The number of second-time movers is 4267.

## Identification strategy

To test the first prediction, I estimate how the average commuting time in the region of residence before the relocation $\overline{C}_{i,t - 1}$ influences the individually chosen commuting time in the target region $C_{i,t = 0}$, I consider a dynamic fixed effects model, where the lag of the dependent variable $C_{i,t - 1}$ is used as an explanatory variable^7^:

2 $$\ln (C_{i,t = 0} ) = \beta_{1} \ln (C_{i,t - 1} ) + \beta_{2} \ln (\overline{C}_{i,t - 1} ) + \beta_{3} X_{i,t}^{\prime } + \mu_{i} + \varepsilon_{i,t}$$

where ${\text{ln}}(C_{i,t = 0} )$ represents the dependent variable, the logarithm of the individual chosen commute in minutes after the relocation *t* = 0, while $\ln (C_{i,t - 1} )$—the lag of the dependent variable—is added as an independent variable. The variable of interest ${\text{ln}}(\overline{C}_{i,t - 1} )$ shows the logarithm of the average commuting time in the region of residence before the relocation *t* − 1. The average commuting time is calculated for each NUTS-3 region and represents the context of previously observed commutes. Further, I include $X_{i,t = 0}^{\prime }$ as a vector of control variables. This vector includes the log wage, calendar years, occupational status and indicator variables for firm size (number of employees, 4 categories), age group (4 categories), occupation (12 categories), industry (9 categories) and region type of the place of residence as well as of the place of work (according to the classification of the Federal Institute for Research on Building, Urban Affairs and Spatial Development BBSR). These region types represent whether individuals live and work in a metropolitan city, city, large town, small town or in a rural area (5 categories). Moreover, $X_{i,t = 0}^{\prime }$ incorporates several dummies indicating whether a worker is a supervisor, has a leading position, is a trained/professional, specialist/expert or has an auxiliary job. In addition, $X_{i,t = 0}^{\prime }$ incorporates a dummy for women, migrants, western Germany and for being low-skilled (without vocational training) medium-skilled (with vocational training) or high-skilled (academic degree). And $\mu_{i}$ shows the time invariant individual-specific effects.

According to prediction 1, $\beta_{2}$ should be positive because individuals with stronger observed commuting backgrounds have a lower disutility of commuting and thus prefer to live outside the city center, thereby facing longer commutes.

However, in the case of *unobserved heterogeneity, omitted variable bias and selectivity* which can influence the estimates of $\overline{C}_{i,t - 1}$ or *sorting—*meaning that movers relocate to certain regions because of their taste for commuting—my results would not be valid. First, to address the issue of unobserved heterogeneity regarding, for example, commuting preferences, the estimates control for individual fixed effects $\mu_{i}$ (Eq. 2). Thus, unobserved heterogeneity regarding individual commuting should not impact my results.

Second, to deal with the issue of omitted variable bias, I conduct several robustness checks excluding observable individual and firm characteristics in my analysis. The results are presented in the robustness checks in Sect. 5 (Table 8) and confirm my presented results, as the results barely change.

Third, workers might endogenously choose whether or not to move. To control for this selectivity, I use a two-stage Heckman selection method (Heckman 1979) where I first account for the decision to move, which can be estimated as a latent variable model:

3 $$P_{i}^{*} = \delta_{1} S_{i} + \varepsilon_{i}$$

With the decision to move:

4 $$P_{i} = \left\{ {\begin{array}{*{20}c} 1 & {if\;\;P_{i}^{*} > 0} \\ 0 & {otherwise} \\ \end{array} } \right.$$

$P_{i}^{*}$ represents the latent variable for the propensity to move between two NUTS-3 regions and $S_{i}$ is a vector of sociodemographic characteristics and information on industry and firm size, which influence individual *i*. To estimate whether or not a worker moves, I use a probit estimation. These results are then taken to construct an inverse Mills ratio. This inverse Mills ratio is then included in the second step equation to correct for selection bias (Eq. 2).

The third issue is sorting: For example, individuals who dislike (like) commuting choose regions with shorter (longer) commuting times. To face this selectivity issue, I include the individual’s own commuting time in the region before the relocation $C_{i,t - 1}$ (see Eq. 2), and perform a robustness check. In line with selectivity, individuals select themselves into a region because of their commuting taste. If people select themselves into regions with longer average commutes because of their taste for long commuting, they should also have commuted longer in the region before the move. To exploit this fact, I perform a reversed regression in which I regress the individual commute in the previous region on the average commuting time in the target region—after the relocation.

5 $${\text{ln}}(C_{i,t - 1} ) = \beta_{0} + \beta_{1} {\text{ln}}(\overline{C}_{i,t = 0} ) + \beta_{2} X_{i,t = 0}^{\prime } + \varepsilon_{i}$$

In line with the above argument indicating selectivity, I should find a positive effect of the average commutes in the destination region $\overline{C}_{i,t = 0}$ on the individuals’ commuting time in the region before the movement $C_{i,t - 1}$. The results are presented in the robustness checks in Sect. 5 (Table 9).

Another neglected effect could be due to imperfect information: when moving to a new region worker have no information about the commuting situation there. Therefore, they might commute longer initially and then change their commutes by relocating again within the new region—thereby explaining the second prediction. However, information about commuting and the local housing market is relatively cheap. Nevertheless, the commuting costs are high: commuting takes time, causes stress, and is very expensive. I would thus expect workers to obtain information about the commuting situation in the new region before they move.

In addition, the decision regarding accommodation might be made under time pressure, thus representing a random event. For example, when individuals have found a new job but then have little time left to find a new apartment. In this case, they might be willing to take any accommodation, wherever it is located, as long as it seems to be acceptable. However, if it appears to be the case that the new commuting time is a random event, first I would not expect the individual’s own previous commuting time as well as the average commuting time in the region before the move to have a significant influence on the selected commuting time in the target region. And second, I would not expect those workers to move again within the new region and adjust their commuting time to the average commuting time in the new region.

The travel time budget—and thus the commuting decision—might also be influenced by trip chaining or by the fraction of remote work. In particular, with the Covid-19-shock remote work has increased and there is some consistency in remote work. Due to the possibility of working from home the travel-time budget becomes more relaxed and thus longer commuting distances might be expected and accepted. However, as my observation period is restricted (2000–2014) and the data does not include the fraction of remote work, I cannot analyze how the results might be affected by the Covid-19-shock. In addition, Brunow and Gründer (2013) found that the daily allocation of time in Germany is affected by trip chaining, such that unobserved factors may influence the time budget. In particular, after migration not just the trip “home-to-work” influences the persistence of habits but also other factors such as shop accessibility or child care institutions leading to a potential bias in estimates. However, I suspect that this bias is negligible in this study, because people living in the destination area still form the daily activity chains.

To test prediction 2, I restrict the sample to workers who move again within the new region, one period after the first move *t* + 1. I use the following identification strategy, in which only changes are analyzed. Because of these differences, individual fixed effects are canceled out:

6 $${\text{ln}}(C_{i,t + 1} - C_{i,t = 0} ) = \beta_{1} ln(\overline{C}_{i,t = 0} - \overline{C}_{i,t - 1} ) + \beta_{2} ln(W_{i,t + 1} - W_{i,t = 0} ) + \varepsilon_{i}$$

The dependent variable $$(C_{i,t + 1} - C_{i,t = 0} )$$ is the change in the individual chosen commuting time after the second and the first move within the new region. The control variable is the change in wages ($$W_{i,t + 1} - W_{i,t = 0} )$$ between the second and the first move. And the key predictor is represented by the difference between the observed commuting time in the new region *t* = 0 and in the region before the move *t* − 1, corresponding to $$(\overline{C}_{i,t = 0} - \overline{C}_{i,t - 1} )$$. This classification of the reference point presupposes that the workers’ perceptions have fully adjusted after one period.

However, this might still not be a correct estimate of the change in the commuting time as workers might endogenously choose whether to move a second time. Therefore, I again use a two-step Heckman selection method (see Eqs. 3, 4). If workers decide to move a second time within the new region, in line with prediction 2, the coefficient $\beta_{1}$ (Eq. 6) should be positive: individuals moving from regions with observed long commutes to a new region (with shorter average commutes) commute too long at first. This leads to a change in the desired commuting durations. Therefore, if they move again within this new region, they reduce their commutes and adopt the commuting behavior prevalent in the new region.

## Empirical analysis of the commuting behavior

### Descriptive statistics

**Fig. 1:**
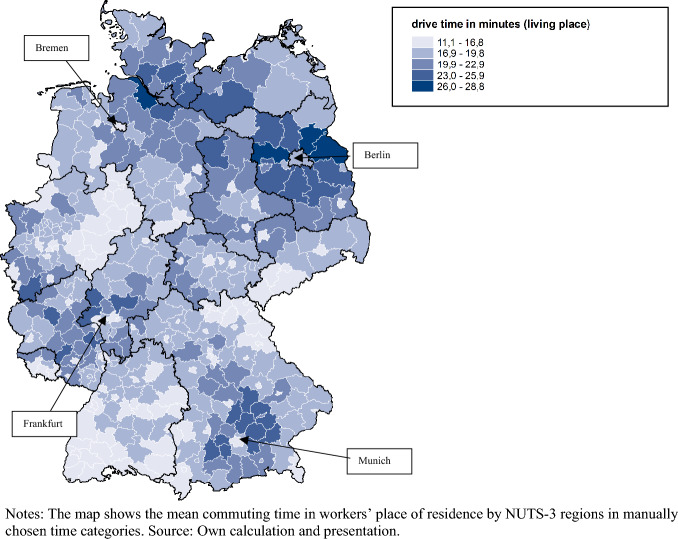
Regional distribution of commuting time in the year 2014

Figure 1 presents the distribution of the average commuting times for the place of residence for each NUTS-3 region in Germany. Workers living in metropolitan cities, like Munich, Berlin, Frankfurt or Bremen, have shorter average commuting times than those in the surrounding regions. Specifically, the average commuting time in metropolitan cities is 16.8 min, while workers in rural areas commute almost 20 min to work on average. This implies that workers who live in large cities are most likely to work there as well, while workers living in the suburbs travel from the surrounding regions into the city center to work. This may be because job opportunities are better in the city center and housing costs are cheaper in the suburbs (Alonso 1964).

#### Comparison of movers and non-movers

To demonstrate how the characteristics of workers who relocate differ from those who do not, I compare the two groups. The results are represented in the Web Appendix A. They show that movers and non-movers differ especially in terms of their productivity-related characteristics: employees who relocate are more highly qualified (academic degree) than non-movers. Differences also become obvious with regard to industries, occupations, and age groups. While the share of movers is much larger between 18 and 34, non-movers are mainly between 35 and 56 years old. Moreover, movers tend to drive an average of 1.2 min longer to work than non-movers. This comparison therefore shows considerable heterogeneity between movers and non-movers.

#### Comparison of movers before and after the relocation

In the following, I examine summary statistics of workers who move. Table 1 shows the difference between movers’ driving times and wages before *t* = − 1 and after the relocation *t* = 0.

**Table 1:** Summary statistics of the daily wage and commuting time

Variable	Mean	Std. dev	25th perc	50th perc	75th perc
Commuting time t = − 1 in minutes	18.8	16.6	6.9	13.9	25.1
$\Delta$ Commuting time t = 0 in minutes	+ 3.9	23.8	− 8.2	2.7	14.9
Wage t = − 1 (euros/day)	85.9	55.9	49.7	74.2	106.5
$\Delta$ Wage t = 0 (euros/day)	+ 12.8	41.0	−2 .7	8.5	27.1
N	15,671				

Means, standard deviation, 25th, 50th, 75th percentiles of commuting time and the wage. Comparison of movers before and after the relocation

The average mover experiences an increase in wages (+ 12.8 euros per day), which supports the idea that workers are more likely to move if they can achieve a wage increase, as non-movers on the other hand only experience an average wage increase of about 3.4 euros per day between two periods. Not only wages rise due to the relocation, the commuting time does so too. On average, the commuting time among movers increases by 3.9 min.

#### Motivation of movers

As already mentioned, when workers move to a new region, they achieve an increase in wages, which could be an important motivation to move. Furthermore, Table 2 shows that 33 percent of workers change their occupation after the move. In addition, almost 34 percent of movers work in a different industry after relocating.

**Table 2:** Summary statistics of changes in occupation, industry, and promotion

Variable	Occupation	Industry	Promotion
Change as a percentage	33.0	33.9	12.7
N	5238	5372	2009

Percentage of workers who change occupation or industry, or are promoted after the relocation t = 0

Workers might therefore move in particular for job-related reasons. Simonsohn (2006) obtains a similar finding. He reports that more than 36 percent of individuals in the US move for job-related reasons. Moreover, in many cases (12.7 percent) the move is associated with a promotion, for example from trained/professional assistant to specialist/expert (see Table 2).

#### Comparison of movers and second-time movers

In the following, I take a closer look at second-time movers. These are workers who relocate a second time within the new region. Table 3 compares these second-time movers with the share of regular movers (workers who move once) after the first and before the second move.

**Table 3:** Summary statistics of main variables

Variable	Movers (%)	Second-time movers (%)
Woman	50.6	47.6
Migrant	3.9	4.3
West Germany	86.4	89.0
Age groups		
18–24	14.5	18.4
25–34	47.9	46.8
35–44	26.8	24.9
45–56	10.8	10.0
Skill level		
Low-skilled	6.5	7.9
Medium-skilled	63.1	68.5
High-skilled	30.4	23.6
N	11,597	4267

Means of main variables. Comparison of movers and second-time movers after the first move t = 0

Of the 15,671 movers in *t* = 0 4,267 relocate a second time in *t* = 1. Especially medium-skilled workers tend to move again within the new region. In addition, the shares of men, migrants, and workers in western Germany are higher for second-time movers, and they are younger on average (between 18 and 24 years old).

Table 4 shows the difference between the daily wages and the commuting times of movers and second-time movers after the first relocation *t* = 0.

**Table 4:** Summary statistics of commuting time and wage

	Variable	Mean	Std. dev	25th perc	50th perc	75th perc	N
Movers	Commuting time (min)	18.7	15.5	7.7	14.6	25.0	11,597
Second-time movers	Commuting time (min)	33.4	22.6	15.2	28.1	48.1	4,267
Movers	Wage (euros/day)	99.7	58.6	61.6	84.1	122.4	11,597
Second-time movers	Wage (euros/day)	96.0	55.0	62.7	82.0	113.8	4,267

Means, standard deviation, 25th, 50th, 75th percentiles of driving time and the wage. Comparison of movers and second-time movers after the first move t = 0

Compared to movers, second-time movers have much longer commuting times after the first move in *t* = 0. Workers who move only once have a commuting time of 18.7 min in *t* = 0, while those who move a second time drive over 14 min longer to work after the first relocation. This results not only from the fact that second-time movers come from regions with longer commutes compared to movers, but also that they are more likely to move from rural regions with longer average commuting times. According to the background context effect, this leads to a higher tolerance for commuting and thus to a longer chosen individual commuting time after the move. This could explain why especially these workers move again within the new region and reduce their commuting time by more than 13 min (see Table 5).

**Table 5:** Summary statistics of commuting time and wage

Variable	Mean	Std. dev	25th perc	50th perc	75th perc
Commuting time t = − 1 in minutes	19.9	17.1	7.6	15.1	26.4
$\Delta$ Commuting time t = 0 in minutes	+ 13.4	27.7	− 2.7	10.7	29.7
$\Delta$ Commuting time t = + 1 in minutes	− 13.5	24.8	− 28.0	− 6.6	2.3
Wage t = − 1 (euros/day)	81.8	51.7	47.4	72.1	101.7
$\Delta$ Wage t = 0 (euros/day)	+ 14.3	36.4	− 0.6	9.2	27.5
$\Delta$ Wage t = + 1 (euros/day)	+ 4.9	29.9	0.2	3.6	9.09
N	4267				

Means, standard deviation, 25th, 50th, 75th percentiles of commuting time and the wage. Comparison of second-time movers before and after the first move and after the second move

Table 5 shows the difference between wages and commuting times before the first move *t* = − 1 after the first move *t* = 0 and after the second move *t* =  + 1 for individuals who moved a second time. As explained above, the increase in the commuting time after the first move is far higher for individuals moving twice than for those moving only once. Second-time movers increase their commuting time by over 13 min in *t* = 0. However, they shorten their commuting time by the same amount after the second relocation in *t* =  + 1. This corrects the originally excessive commuting time, and confirms prediction 2.

### Empirical analysis

**Prediction 1: the average commuting time in the region a person leaves has a positive influence on the individually selected commuting time in the destination region**

In the following, I test the first prediction, in which I investigate how the average commuting time in the region before the relocation influences the individually selected commuting time in the target region (Eq. 2). As workers may endogenously choose to move, I use a two-step regression (Heckman 1979). In the first step I estimate a probit regression for the decision to relocate (Eq. 3). The results for this probit regression are provided in the Web Appendix B and show, for example, that workers with higher wages, high-skilled workers and workers in western Germany are more likely to relocate. In the second step, I use the inverse Mill’s ratio from the first step as an additional control variable and analyze how the average commuting time in the region before the relocation influences the commuting time in the new region (Eq. 2). Table 6 shows the results of 4 specifications.

**Table 6:** Individually selected commuting time after relocation

NUTS-3 region	Dependent variable: ln($C_{i,t = 0}$)			
	Model 1	Model 2	Model 3	Model 4
Ln($C_{i,t - 1}$)	0.228*** (0.006)	0.225*** (0.006)	0.225*** (0.006)	0.225*** (0.006)
Ln($\overline{C}_{i,t = - 1}$)		0.216*** (0.030)	0.222*** (0.029)	0.212*** (0.029)
Inverse of Mill’s ratio*	0.620*** (0.205)	0.560*** (0.206)	0.143 (0.144)	0.531*** (0.206)
Ln(wage)	0.107*** (0.034)	0.100*** (0.034)		0.117*** (0.034)
Ln($$wage_{t - 1} )$$				− 0.103*** (0.015)
Medium-skilled	0.172*** (0.047)	0.163*** (0.047)	0.103** (0.042)	0.165*** (0.047)
High-skilled	0.231*** (0.079)	0.211*** (0.079)	0.093 (0.066)	0.206*** (0.079)
Migrant	− 0.106 (0.066)	− 0.098 (0.066)	− 0.044 (0.063)	− 0.092 (0.066)
Specialist/expert	0.036 (0.044)	0.035 (0.044)	0.032 (0.044)	0.037 (0.044)
Trained/professional assistant	0.003 (0.038)	0.002 (0.038)	0.004 (0.038)	0.005 (0.038)
Age groups	Yes	Yes	Yes	Yes
Occupation dummies	Yes	Yes	Yes	Yes
Industry dummies	Yes	Yes	Yes	Yes
Occupational status	Yes	Yes	Yes	Yes
Firm size (Number of workers)	Yes	Yes	Yes	Yes
Year dummies	Yes	Yes	Yes	Yes
Place of residence type	Yes	Yes	Yes	Yes
Place of work type	Yes	Yes	Yes	Yes
Constant	− 0.667 (0.830)	− 1.030 (0.827)	0.841* (0.508)	− 0.560 (0.830)
N	45,232	45,232	45,232	45,232
N (cluster)	15,262	15,262	15,262	15,262
*R*^2^	0.5773	0.5777	0.5775	0.5783
Adj. *R*^2^	0.3607	0.3614	0.3611	0.3622

The table reports regressions of the individually selected log commuting times after the first relocation on the average log commuting time in the region before the relocation and control variables. Standard errors clustered by individuals, below parameter estimates. Levels of significance: *1%, **5%, ***10%

*Inverse of Mill’s ratio is obtained from the first stage probit estimation of the move

According to model 1, which includes the lag of the individual commuting time *t* − 1, the longer the commuting time was in the region before the relocation, the longer the individually selected commuting time is in the target region. In addition, the wage has a positive significant effect, which might be the result of compensatory wages for longer commutes as shown by Mulalic et al. (2014). In the second model I include the average commuting time in the region in which the previous place of residence was located $\overline{C}_{i,t - 1}$ as a proxy for commuting options observed in the past. Consistent with the first prediction, model 2 shows a positive significant effect on the individual commuting time. Moreover, the effect can be interpreted as causal, as I control for selectivity and unobserved heterogeneity, and can rule out the issue of omitted variable bias and sorting (see Sect. 5.3). Hence, mobile workers coming from NUTS-3 regions with longer observed commutes have a greater tolerance for commuting and choose longer individual commutes in the target region. This indicates the presence of a context effect and is therefore consistent with the result obtained by Simonsohn (2006). However, a comparison of the effects with those found by Simonsohn (2006) shows that he overestimates the effect of the context (see Sect. 5.3 Table 8). This is because he does not include individual unobserved fixed effects. In addition, comparing $R^{2}$ reveals that the model I consider performs much better than that of Simonsohn (2006) (0.36 vs. 0.15).

Since commuting may be endogenous with respect to wages, model 4 excludes daily wages, which has little impact on the size of the coefficient of $\overline{C}_{i,t - 1}$. In addition, in model 5 I include time-lagged wages *t* − 1. In this estimation, too, the result shows no change for the variable of interest $\overline{C}_{i,t - 1}$.

Thus, the results indicate that workers’ current commuting behavior is affected not only by their own previous commuting time but also by the average commuting time in the region they moved from.

**Prediction 2: Individuals readjust their commuting times and move again when remaining in the new region**

If workers relocate from regions with longer commutes to regions with shorter average commuting times ($\overline{C}_{i,t - 1}$ > $\overline{C}_{i,t = 0}$), they initially commute longer than the average in the target region. The reason for this is that they have a greater tolerance for commuting as they come from regions where long commutes are common. Nevertheless, if they remain in the new region and observe fewer commutes, they become dissatisfied with their initially chosen commutes and their desired commuting time changes. Therefore, I expect them to reduce their commutes by relocating again within the new region. To analyze the adjustment of the commuting time after a second move, I consider only individuals who move again within one year after relocating to the new region. A total of 4,135 individuals move again within the new NUTS-3 region in *t* = 1.

The regression estimates of Eq. 6 are presented in Table 7, where $(C_{i,t + 1} - C_{i,t = 0} )$, the dependent variable, measures the change in the individual commuting time after the second and the first relocation. Therefore, it represents the adjustment of the individual commuting time between *t* = 0 and *t* =  + 1. The key predictor is the difference between the average commuting time in the new region and that in the previous region $(\overline{C}_{i,t = 0} - \overline{C}_{i,t - 1} )$. Moreover, as workers may endogenously choose whether to move a second time, I use a two-step regression (Heckman 1979): in the first step, I estimate a probit regression for the decision to relocate a second time in the new region (Eq. 3). The results of this probit regression can be found in the Web Appendix C. They show, for example, that the greater the difference between the average commuting time and the individual’s own selected commuting time in the target region, the more likely a second move is. In the second step, I use the inverse Mill’s ratio from the first step as an additional control variable. The results are presented in Table 7 and are seen to be in line with prediction 2, the greater the difference between the new and the old region $(\overline{C}_{i,t = 0} - \overline{C}_{i,t - 1} )$ the stronger the adjustment of the individually chosen commuting time after the second move is. Comparing the estimated effect of $\beta_{2}$ (Table 7) with the estimation of $\beta_{2}$ in prediction 1 (Table 6 model 2) it can be seen that the coefficient $\beta_{2}$ of the first prediction is twice as large as $\beta_{2}$ in the second prediction. Thus, second-time movers do not fully reverse the original impact of $\overline{C}_{i,t - 1}$, but it is moving in that direction.

**Table 7:** Adjustment of the commuting time in t + 1

NUTS-3 region	Dependent variable: ln($C_{i,t + 1} - C_{i,t = 0}$)
${\text{Ln}}(\overline{C}_{i,t = 0} - \overline{C}_{i,t - 1} )$	0.100* (0.046)
Change in ln(wage)	0.049 (0.108)
Inverse of Mill’s ratio*	1.971*** (0.084)
Constant	− 2.729*** (0.084)
N	4,135
*R*^2^	0.3531
Adj. *R*^2^	0.3526

The table reports the regression of the adjustment of the individually selected commuting time after the second move on the difference between the average commutes in the new and the old region. Standard errors clustered by NUTS-3 regions, below parameter estimates. Levels of significance: *1%, **5%, ***10%

*Inverse of Mill’s ratio is obtained from the first stage probit estimation of moving again within the new region

With this result, I can therefore rule out an explanation for the commuting behavior that is based on stable unobserved differences across movers from different regions, as individuals readjust their commuting time by moving again within the new region—they adopt the commuting behavior of the new region.

### Robustness checks

Although the presence of stable unobserved differences can be ruled out by confirming prediction 2, there could be other explanations for the presented results and several issues that might influence the outcome, such as unobserved heterogeneity, omitted variable bias, selectivity, and sorting. However, in the following, I am able not only to reject other explanations, but also to confirm my results by means of several robustness checks. Therefore, the effect of $\overline{C}_{i,t - 1}$ on $C_{i,t = 0}$ can be interpreted as causal.

#### Unobserved heterogeneity

In fact, unobserved heterogeneity can have an influence on the estimates of $\overline{C}_{i,t - 1}$, thereby driving the effect of the context (see Sect. 4). To deal with this issue, I include individual fixed effects in my analysis (see Eq. 2). This is especially important, and failure to do so generates a bias. This can be observed in Table 8 (model 1). Excluding individual fixed effects overestimates the effect of the individual previous commuting time $C_{i,t - 1}$, and underestimates the influence of the context of previously observed commutes $\overline{C}_{i,t - 1}$. It is therefore important to include individual fixed effects. Failure to do so leads to a bias, as in the study by Simonsohn (2006) which does not include individual fixed effects in the analysis and therefore underestimates the effect of the context.

**Table 8:** Robustness check: individually selected commuting time after the move

NUTS-3 region	Dependent variable: ln($C_{i,t = 0}$)				
	Model 1	Model 2	Model 3	Model 4	Model 5
Ln($C_{i,t - 1}$)	0.531*** (0.004)	0.226*** (0.006)	0.226*** (0.006)	0.227*** (0.006)	0.225*** (0.006)
Ln($\overline{C}_{i,t = - 1}$)	0.154*** (0.023)	0.224*** (0.029)	0.220*** (0.029)	0.223*** (0.029)	0.224*** (0.029)
Inverse of Mill’s ratio*	0.092 (0.078)	− 0.009 (0.032)	0.292** (0.132)	− 0.021 (0.032)	
Ln(wage)	0.061*** (0.013)		0.088*** (0.027)		0.034 (0.024)
Medium-skilled	0.039** (0.018)		0.119*** (0.040)		0.078** (0.035)
High-skilled	0.035 (0.028)		0.124** (0.062)		0.040 (0.048)
Migrant	− 0.037* (0.019)		− 0.063 (0.063)		− 0.027 (0.060)
Specialist/expert	0.026 (0.024)		0.016 (0.044)		0.027 (0.044)
Trained/professional assistant	0.001 (0.021)		− 0.012 (0.038)		0.001 (0.038)
Age groups	Yes		Yes		Yes
Occupation dummies	Yes		Yes		Yes
Industry dummies	Yes	Yes			Yes
Occupational status	Yes		Yes		Yes
Firm size (Number of workers)	Yes	Yes			Yes
Year dummies	Yes	Yes	Yes	Yes	Yes
Place of residence type	Yes	Yes	Yes	Yes	Yes
Place of work type	Yes	Yes	Yes	Yes	Yes
Constant	0.190 (0.310)	1.438*** (0.134)	− 0.042 (0.533)	1.476*** (0.132)	1.194*** (0.139)
N	45,232	45,232	45,232	45,232	45,232
N (cluster)	15,262	15,262	15,262	15,262	15,262
*R*^2^	0.3415	0.5768	0.5763	0.5753	0.5776
Adj. *R*^2^	0.3407	0.3606	0.3595	0.3586	0.3612

The table reports regressions of the individually selected log commuting times after the first relocation on the average log commuting time in the region before the move and control variables. Standard errors clustered by individuals, below parameter estimates. Levels of significance: *1%, **5%, ***10%

*Inverse of Mill’s ratio is obtained from the first stage probit estimation of the move

#### Omitted variable bias

In addition, I conduct several robustness checks excluding individual and firm characteristics. In model 2 (Table 8) I exclude firm characteristics, which yields similar results for the context of previously observed commutes to those in Table 6 (model 2), which included all control variables. Also, almost the same results are obtained when firm characteristics are excluded and when both individual and firm characteristics are excluded (models 3 and 4). Thus, the results on the previous average commuting time are very robust and do not seem to be influenced by observed individual or firm characteristics. This leads me to conclude that there is no evidence of omitted variable bias.

#### Selectivity

To control for the selectivity of a relocation—as workers may endogenously choose to relocate—I use a two-step Heckman selection model (Heckman 1979), in which I control for the selectivity of a relocation (Eq. 3). To gain an impression of whether selectivity is important I estimate the model without controlling for selectivity. The results are provided in Table 8 (model 5) and show almost the same effects for previously observed commutes as those in Table 6 (model 2). Only the coefficients for wages and the skill-level variables change. Thus, controlling for the selectivity of the relocation is not important for interpreting the variable of interest but influences other control variables.

#### Sorting

Another issue might be sorting, as workers select themselves into certain regions because of their taste for commuting. To address this issue, I run a reversed regression of Eq. 5. In line with the definition of sorting, I should find a positive correlation between the average commuting time in the destination region and the individual commuting time in the region before the move. However, my results show no significant effect of the average commuting time in the destination regions (Table 9).

**Table 9:** Robustness check: individuals select themselves into regions because of their taste for commuting

NUTS-3 region	Dependent variable ln($C_{i,t - 1}$)
Ln($C_{i,t = 0}$)	0.082*** (0.008)
Ln($\overline{C}_{i,t = 0}$)	− 0.109 (0.077)
Ln($\overline{C}_{i,t - 1}$)	0.951*** (0.059)
Ln(wage)	0.086*** (0.021)
Medium-skilled	0.049* (0.029)
High-skilled	0.078** (0.034)
Migrant	− 0.109** (0.043)
Specialist/expert	0.053 (0.047)
Trained/professional assistant	− 0.005 (0.042)
Woman	− 0.070*** (0.019)
Age groups	Yes
Occupation dummies	Yes
Industry dummies	Yes
Occupational status	Yes
Firm size (Number of workers)	Yes
Year dummies	Yes
Place of residence type	Yes
Place of work type	Yes
Constant	− 0.658** (0.297)
N	15,262
$R^{2}$	0.056
Adj.$R^{2}$	0.0520

The table reports the regression of the individual commuting time in the previous region on the average commuting time in the target region (after the relocation). Standard errors clustered by NUTS-3 regions, below parameter estimates. Levels of significance: *1%, **5%, ***10%

Thus, there is no sign of a sorting process—individuals do not select themselves into regions because of their taste for commuting—but this once again shows the presence of the context effect.

Moreover, workers might also move for job-related reasons, such as higher wages. As wages are highly correlated with commuting in theory, I consider only workers who earn almost the same wage before and after the first relocation.^8^ Table 10 shows that the average commuting time in the region before the move has a positive and significant influence on the commuting time of workers who do not achieve an increase in wages after the relocation. This indicates that endogeneity issues with respect to wages do not drive the results. In addition to restricting the sample to persons earning the same wage before and after relocating, I also restrict it to workers who do not change their task level. Once again, the coefficient of the average commuting time in the previous region does not change.

**Table 10:** Robustness check: movers, who earn almost the same wage before and after relocating (1) and who have the same wage as well as the same task level (2) before and after relocating

NUTS-3 region	(1) Dependent variable ln($C_{i,t = 0}$)	(2) Dependent variable ln($C_{i,t = 0}$)
Ln($C_{i,t - 1}$)	0.202*** (0.013)	0.199*** (0.015)
Ln($\overline{C}_{i,t = - 1}$)	0.381*** (0.071)	0.361*** (0.081)
Ln(wage)	− 0.164* (0.088)	− 0.125 (0.109)
Medium-skilled	− 0.089 (0.102)	− 0.073 (0.117)
High-skilled	− 0.033 (0.151)	− 0.042 (0.196)
Migrant	0.161 (0.108)	0.060 (0.138)
Specialist/expert	0.066 (0.103)	0.014 (0.197)
Trained/professional assistant	0.063 (0.081)	0.018 (0.155)
Age groups	Yes	Yes
Occupation dummies	Yes	Yes
Industry dummies	Yes	Yes
Occupational status	Yes	Yes
Firm size (Number of workers)	Yes	Yes
Year dummies	Yes	Yes
Place of residence type	Yes	Yes
Place of work type	Yes	Yes
Constant	1.745*** (0.449)	1.687*** (0.559)
N	9,193	7,473
N (cluster)	3,094	2,514
*R*^2^	0.5797	0.5833
Adj. *R*^2^	0.3603	0.3645

The table reports regressions of the individually selected log commuting time after the first relocation on the average log commuting time in the region before the move and control variables. Standard errors clustered by individuals, below parameter estimates. Levels of significance: *1%, **5%, ***10%

To sum up, the robustness checks show that it is crucial to include the individual fixed effects when investigating the individual commuting behaviors. In addition, the robustness checks indicate that my results on the average commuting time are not driven by omitted variable bias—as the coefficient is very robust when individual and firm-specific characteristics are excluded. Furthermore, sorting does not seem to influence my results, either. Therefore, the investigated influence of the previously observed commutes on the individually chosen commuting time (Table 6) can be interpreted as causal.

### Effect heterogeneity

In the following, I investigate the heterogeneous effects of the context on the individual selected commuting time. I differentiate movers by different age groups, skill levels, and gender. In addition, I consider movers between different types of regions—urban and rural areas—as well as movers between labor market regions.

#### Age groups, gender, and skill level

Since it is possible that individuals differ in their behavior due to their age, gender, or skill level, I take up this point by performing the estimation for different interactions (Web Appendix D). In particular, I interact the average commuting time in the region before the relocation $\overline{C}_{i,t = - 1}$ with age, gender, and skill level. The results show no significant group differences in terms of age and skill level. Nor can any significant differences be observed between women and men. Thus, there is no effect heterogeneity for different groups regarding age categories, skill level, or gender.

#### Movers between different types of rural and urban regions

Considering movers between different types of place of residence, I interact the average commuting time in the previous location $\overline{C}_{i,t = - 1}$ with the different types of rural and urban regions before and after the move.^9^ The results are shown in the Web Appendix E and indicate that the effect of the context of previously observed commutes is strongest for those moving to urban areas, especially for the group moving from a rural to an urban area.^10^ This is related to the fact that workers who previously lived in a rural area with long average commutes are used to commuting long distances. Therefore, when moving to urban regions such workers have a higher tolerance for commuting and choose longer than average commutes in the urban region. However, for movers to rural areas the results indicate a smaller or insignificant effect of the context. The reason could be that the majority of workers moving from urban to rural areas do not only relocate their place of residence but also take up a new job in the rural area. Thus, other conditions, such as job availability, are more important than commuting preferences for this group of movers.

Hence, the results indicate that the size of the effect of the context depends particularly on the region type of the place of residence before and after the relocation. Considering only movers between metropolitan areas (Simonsohn 2006) might therefore lead to a bias in the estimated effect.

#### Labor market regions

Next, I show the results for individuals moving between German labor market regions (Kosfeld and Werner 2012). The restrictions are the same as for movers between NUTS-3 regions, i.e., workers have to relocate both their place of work and their place of residence to a different German labor market region. Moreover, the labor market region of the place of work and the place of residence must be constant for two years before and after the move. In contrast to the consideration of individuals moving between NUTS-3 regions, I calculate the average commuting time at the level of labor market regions (as a proxy for previously observed commuting options). The results are shown in Web Appendix F and are comparable with the effect of the context for persons moving between NUTS-3 regions (Table 6).

## Conclusion

This study investigates for the first time commuting behavior in terms of a behavioral economic concept based on geo-referenced data for Germany. The basis of this investigation is the approach developed by Simonsohn (2006), who examines commuting behavior for the US. However, I can show that his estimated effects are biased due to the absence of individual fixed effects and the consideration only of individuals moving between metropolitan areas.

The presented results show that workers’ commuting decisions are influenced by commuting options observed in the past. This explains why individuals who move from different regions to one and the same region initially commute differently: individuals moving from areas with long average commutes have a greater tolerance for commuting and therefore commute more than individuals coming from regions with shorter commutes. However, if they remain in the new region, they adjust their initially chosen commuting times to the average commutes in the new region. This refutes the assumption of stable unobserved differences across individuals. Instead, individuals change their marginal utility of commuting when moving to a new region, as they adjust their commuting time by means of a second relocation within the new region. The reason for this behavior is the change in the context: the original context was seen as the average commuting time in the previous region, but the context changes with the relocation to a new region. Thus, commuting preferences change. In addition, the results indicate that selectivity and sorting do not influence the effect of the context, but it is crucial to include individual fixed effects. Moreover, the context has different effects depending on the region type of the place of residence: the context effect is greatest for those moving from rural to urban areas.

However, the travel time budget can be influenced by remote work that increased during the Covid-19-shock and might increase the expected and acceptable commuting distance. Future research could examine whether such increase in remote work influences the effect of the context. Additionally, for future investigation that examine consumer preferences and other labor market decisions, the study highlights the importance of identifying the context of previously observed options and including them in the analysis. Finally, the results indicate the essentiality of including individual fixed effects, as they influence the outcome of commuting decisions.

## Supplementary Information

Below is the link to the electronic supplementary material.

Supplementary file1 (DOCX 29 kb)

## Footnotes

1 In contrast, Simonsohn (2006) uses the average commuting time on the city level, as he only analyzes movers between two metropolitan areas. Thus, while the predictions are quite similar to those of Simonsohn (2006), the objects of investigation differ due to the different target group of movers.

2 However, the results obtained by Simonsohn (2006) show that the context has almost the same effect for people who use public transport.

3 For the analysis in this study I consider commuting time. However, all the results are very similar when commuting distance is used.

4 In a robustness check, I investigate the effect of the context for this group, then also provide evidence of a context effect for this group of movers.

5 Estimating the model for the group of movers who only change their place of residence also reveals an effect of the context. The results can be provided additionally on request.

6 However, investigating movers between German labor market regions generates almost the same results (see Web Appendix G).

7 In this sample, I include all workers who relocate between two NUTS-3 regions. For all workers I have 5 observations, two observations before the move, the period of the relocation, and two after.

8 Wages are rounded to the nearest ten.

9 I consider only the location of the place of residence.

10 However, as I cannot take into account dense traffic or congestion when calculating the commuting time, the results might underestimate the commuting costs especially in dense urban areas.
