# Supplementary material for: Persistence of commuting habits: context effects in Germany
Source: Ann Reg Sci. 2023 May 26:1–26. Online ahead of print. doi: 10.1007/s00168-023-01223-4 (PMC10213587; doi:10.1007/s00168-023-01223-4)
Supplement: Supplementary file 1 — Supplementary file1 (DOCX 29 kb) [file 168_2023_1223_MOESM1_ESM.docx]

Appendix for Web Publication

A. Summary statistics of main variables for movers before the movement and workers who do not move.

| Variable | Movers | Non-movers |
| --- | --- | --- |
| Woman | 49.8% | 45.5% |
| Migrant | 4.0% | 6.0% |
| West | 87.1% | 81.5 % |
| Age groups |  |  |
| 18-24 | 15.5% | 10.7% |
| 25-34 | 47.6% | 23.0% |
| 35-44 | 26.3% | 31.3% |
| 45-56 | 10.6% | 35.1% |
| Education |  |  |
| Low-skilled | 15.8% | 15.1% |
| Medium-skilled | 64.6% | 72.2 % |
| High-skilled | 28.6% | 12.7 % |
| Wage (euro/day) | 85.9 (55.9) | 86.7 (55.1) |
| Commuting time in minutes | 18.8 (16.6) | 17.6 (14.7) |
| Industry |  |  |
| Primary sector | 2.0% | 3.0% |
| Food manuf. | 2.1% | 2.4% |
| Consumer goods | 2.0% | 2.6% |
| Industrial goods | 6.0% | 6.0% |
| Capital goods | 9.2% | 11.6% |
| Construction | 3.3% | 6.0% |
| Personal services | 21.9% | 19.4% |
| Business services | 27.2% | 21.5% |
| Public sector | 26.3% | 24.1% |
| Occupation |  |  |
| Agricultural workers | 0.8% | 1.2% |
| Lower manual occupations | 5.9% | 12.3% |
| Higher manual occupations | 8.4% | 14.9% |
| Technicians | 5.2% | 5.2% |
| Engineers | 6.4% | 3.0% |
| Lower services | 7.8% | 11.6% |
| Higher services | 7.0% | 5.8% |
| Semi-Professionals | 11.4% | 9.3% |
| Professionals | 5.5% | 2.0% |
| Lower administrative occupations | 7.7% | 8.1% |
| Higher administrative occupations | 28.0% | 22.9% |
| Managers | 5.4% | 3.1% |
| Supervisor | 2.5% | 2.1% |
| Leading position | 0.2% | 0.7% |
| Specialist/expert | 30.4% | 20.4% |
| Trained/professional assistant | 64.4% | 71.2% |
| auxiliary activity | 2.5% | 6.4% |
| Firm size |  |  |
| 0-9 | 12.9% | 13.4% |
| 10-49 | 25.8% | 24.6% |
| 50-249 | 28.6% | 28.7% |
| 250-499 | 10.3% | 10.9% |
| >499 | 22.4% | 22.3% |
| N | 15,671 | 18,002,997 |

Notes: Means and standard deviation (in parentheses) of main variables. Comparison of movers and non-movers before the movement in t=-1.

B.: Probit regression whether workers move in t=0 (first movement)

| NUTS-3 region | Worker relocate in t=0 |
| --- | --- |
| Ln(wage) | 0.113*** |
|  | (0.007) |
| Woman | 0.067*** |
|  | (0.006) |
| High-skilled | 0.334*** |
|  | (0.014) |
| Medium-skilled | 0.160*** |
|  | (0.011) |
| Migrant | -0.139*** |
|  | (0.017) |
| Supervisor | 0.042** |
|  | (0.019) |
| Leading position | 0.058** |
|  | (0.028) |
| Specialist/expert | 0.018 |
|  | (0.015) |
| Trained/professional assistant | 0.009 |
|  | (0.013) |
| West Germany | 0.116*** |
|  | (0.014) |
| Age groups | Yes |
| Occupation dummies | Yes |
| Industry dummies | Yes |
| Occupational status | Yes |
| Firm size (Number of workers) | Yes |
| Year Dummies | Yes |
| Residence place type | Yes |
| Working place type | Yes |
| Constant | -5.443*** |
|  | (0.115) |
| N | 17,789,084 |

Notes: The table reports the results of the probit regression. whether a worker moves in t=0 (first step of the Heckman selection model). Standard errors clustered by NUTS-3 regions. below parameter estimates. Levels of significance: *1%. **5%. ***10%.

C.: Probit regression whether workers move for a second time.

| NUTS-3 region | Workers moves for a second time |
| --- | --- |
| Ln($\bar{C}_{i.t=0}$) | 0.237 |
|  | (0.246) |
| Ln($C_{i.t=0}-\bar{C}_{i.t=0}$) | 0.502*** |
|  | (0.044) |
| Ln(wage) | -0.029 |
|  | (0.040) |
| Woman | -0.086*** |
|  | (0.032) |
| High skilled | -0.246*** |
|  | (0.054) |
| Medium skilled | -0.058 |
|  | (0.037) |
| Migrant | 0.132** |
|  | (0.064) |
| Supervisor | -0.046 |
|  | (0.067) |
| Leading position | 0.087 |
|  | (0.111) |
| Specialist/expert | -0.030 |
|  | (0.094) |
| Trained/professional assistant | -0.020 |
|  | (0.076) |
| West | 0.154*** |
|  | (0.028) |
| Age groups | Yes |
| Occupation dummies | Yes |
| Industry dummies | Yes |
| Occupational status | Yes |
| Firm size (Number of workers) | Yes |
| Year Dummies | Yes |
| Residence place type | Yes |
| Working place type | Yes |
| Constant | -1.211 |
|  | (0.824) |
| N | 15,262 |

Notes: The table reports the results of the probit regression. whether a worker moves for a second time (first step of the Heckman selection model). Standard errors clustered by NUTS-3 regions. below parameter estimates. Levels of significance: *1%. **5%. ***10%.

D: Robustness check: Individually selected commuting time after the movement with different interaction effects.

| NUTS-3 region | Model 1 | Model 2 | Model 3 |
| --- | --- | --- | --- |
| Ln($C_{i.t-1}$) | 0.225*** | 0.225*** | 0.225*** |
|  | (0.006) | (0.006) | (0.006) |
| Ln($\bar{C}_{i.t=-1}$) | 0.224*** | 0.194*** | 0.263** |
|  | (0.081) | (0.039) | (0.113) |
| Inverse of Mill´s ratio* | 0.559*** | 0.561*** | 0.556*** |
|  | (0.206) | (0.206) | (0.206) |
| Ln(wage) | 0.099*** | 0.100*** | 0.099*** |
|  | (0.034) | (0.034) | (0.034) |
| Ln($\bar{C}_{i.t-1}$)##Education |  |  |  |
| …medium skilled |  |  | -0.064 |
|  |  |  | (0.119) |
| …high skilled |  |  | -0.019 |
|  |  |  | (0.123) |
| Medium skilled | 0.163*** | 0.163*** | 0.346 |
|  | (0.047) | (0.047) | (0.340) |
| High skilled | 0.211*** | 0.211*** | 0.265 |
|  | (0.079) | (0.079) | (0.358) |
| Migrant | -0.098 | -0.098 | -0.097 |
|  | (0.066) | (0.066) | (0.066) |
| Specialist/expert | 0.034 | 0.034 | 0.034 |
|  | (0.044) | (0.044) | (0.044) |
| Trained/professional assistant | 0.002 | 0.002 | 0.002 |
|  | (0.038) | (0.038) | (0.038) |
| Log ($\bar{C}_{i.t=-1}$)##women |  | 0.045 |  |
|  |  | (0.055) |  |
| Log ($\bar{C}_{i.t=-1}$)##Age groups |  |  |  |
| …25-34 | -0.198 |  |  |
|  | (0.254) |  |  |
| …35-44 | -0.140 |  |  |
|  | (0.282) |  |  |
| …older than 44 | 0.010 |  |  |
|  | (0.354) |  |  |
| Age groups | Yes | Yes | Yes |
| Occupation dummies | Yes | Yes | Yes |
| Industry dummies | Yes | Yes | Yes |
| Occupational status | Yes | Yes | Yes |
| Firm size (Number of workers) | Yes | Yes | Yes |
| Year Dummies | Yes | Yes | Yes |
| Residence place type | Yes | Yes | Yes |
| Working place type | Yes | Yes | Yes |
| Constant | -0.896 | -1.035 | -1.148 |
|  | (0.808) | (0.827) | (0.878) |
| N | 45.232 | 45.232 | 45.232 |
| N (cluster) | 15.262 | 15.262 | 15.262 |
| $R^{2}$ | 0.5777 | 0.5777 | 0.5777 |
| Adj. $R^{2}$ | 0.3614 | 0.3614 | 0.3613 |

Notes: The table reports regressions of the individually selected log commuting time after the first relocation on the average log commuting time of the region before the movement and control variables. Standard errors clustered by individuals. below parameter estimates. Levels of significance: *1%. **5%. ***10%.

*Inverse of Mill´s ratio is obtained from the first stage probit estimation of the movement.

E: Robustness check: Relocation between different types of regions.

| NUTS-3 region | Dependent variable ln($C_{i.t=0}$) |
| --- | --- |
| Ln($C_{i.t-1}$) | 0.226*** |
|  | (0.006) |
| Ln($\bar{C}_{i.t=-1}$) | 0.218*** |
|  | (0.066) |
| Ln($\bar{C}_{i.t=-1}$)##rural to rural | -0.134* |
|  | (0.080) |
| Ln($\bar{C}_{i.t=-1}$)##urban to rural | -0.368*** |
|  | (0.087) |
| Ln($\bar{C}_{i.t=-1}$)##rural to urban | 0.510*** |
|  | (0.090) |
| Inverse of Mill´s ratio* | 0.570*** |
|  | (0.206) |
| Ln(wage) | 0.101*** |
|  | (0.034) |
| Medium skilled | 0.162*** |
|  | (0.047) |
| High skilled | 0.209*** |
|  | (0.079) |
| Dummy migrant | -0.096 |
|  | (0.066) |
| Specialist/expert | 0.035 |
|  | (0.044) |
| Trained/professional assistant | 0.003 |
|  | (0.038) |
| Age groups | Yes |
| Occupation dummies | Yes |
| Industry dummies | Yes |
| Occupational status | Yes |
| Firm size (Number of workers) | Yes |
| Year Dummies | Yes |
| Residence place type | Yes |
| Working place type | Yes |
| Constant | -1.064 |
|  | (0.829) |
| N | 45.232 |
| N (cluster) | 15.262 |
| $R^{2}$ | 0.5787 |
| Adj. $R^{2}$ | 0.3628 |

Notes: The table reports regressions of the individually selected log commuting time after the first relocation on the average log commuting time of the region before the movement and control variables. Standard errors clustered by individuals below parameter estimates. Levels of significance: *1%. **5%. ***10%.

*Inverse of Mill´s ratio is obtained from the first stage probit estimation of the movement.

F. Robustness check: Relocation between German labour market regions

| Labor market region level | Dependent variable ln($C_{i.t=0}$) |
| --- | --- |
| Ln($C_{i.t-1}$) | 0.198*** |
|  | (0.006) |
| Ln($\bar{C}_{i.t=-1}$) | 0.217*** |
|  | (0.040) |
| Inverse of Mill´s ratio* | 0.562*** |
|  | (0.214) |
| Ln(wage) | 0.095*** |
|  | (0.035) |
| Medium skilled | 0.135*** |
|  | (0.052) |
| High skilled | 0.182** |
|  | (0.088) |
| Migrant | -0.155** |
|  | (0.074) |
| Specialist/expert | -0.019 |
|  | (0.048) |
| Trained/professional assistant | -0.035 |
|  | (0.041) |
| Age groups | Yes |
| Occupation dummies | Yes |
| Industry dummies | Yes |
| Occupational status | Yes |
| Firm size (Number of workers) | Yes |
| Year Dummies | Yes |
| Residence place type | Yes |
| Working place type | Yes |
| Constant | -0.920 |
|  | (0.862) |
| N | 40.427 |
| N (cluster) | 13.681 |
| $R^{2}$ | 0.5593 |
| Adj. $R^{2}$ | 0.3323 |

Notes: The table reports regressions of the individually selected log commuting time after the first relocation on the average log commuting time of the region before the movement and control variables. Standard errors clustered by individuals below parameter estimates.: *1%. **5%. ***10%.

*Inverse of Mill´s ratio is obtained from the first stage probit estimation of the movement.
